# SiO_2_ and ZnO nanoparticles and salinity stress responses in hydroponic lettuce: selectivity, antagonism, and interactive dynamics

**DOI:** 10.3389/fpls.2025.1634675

**Published:** 2025-09-26

**Authors:** Chungkeun Lee, Seunghyun Choi, Daniel I. Leskovar

**Affiliations:** Texas A&M AgriLife Research and Extension Center, Texas A&M University, Uvalde, TX, United States

**Keywords:** deep water culture, controlled environment agriculture, NaCl, CaCl_2_, antioxidant capacity, eustress, phytotoxicity

## Abstract

Salinity stress negatively affects plant growth but can also act as a eustressor, enhancing nutraceutical quality. Nanoparticles (NPs) have unique physical and chemical properties that can impact crop growth and abiotic stress responses in both beneficial and detrimental ways. This study investigated the potential of SiO_2_ and ZnO NPs to alleviate salinity stress or enhance nutraceutical quality by synergizing with the eustressor effects of salinity in hydroponically grown lettuce. Two-week-old lettuce seedlings (*Lactuca sativa* cv. Green Forest) were transplanted into a 5-L deep water culture system and grown for four weeks in a customized growth chamber set at 25 °C with 230 µmol/m^2^/s photosynthetic photon flux density (PPFD). The nutrient solution was maintained at an electrical conductivity (EC) of 1.5 dS/m and pH 5.8, and replenished weekly. A factorial design was employed with four salinity treatments (non-saline, 50 mM NaCl, 33.3 mM CaCl_2_, and 25 mM NaCl + 16.6 mM CaCl_2_) and three nanoparticle treatments (no-NP control, 100 ppm SiO_2_, and 100 ppm ZnO). Overall, NPs improved lettuce growth under non-saline conditions. Specifically, SiO_2_ NPs increased shoot and root biomass, root system architecture, and antioxidant enzyme activities (superoxide dismutase-SOD and glutathione reductase-GR) compared to controls, while ZnO NPs improved root biomass and architecture, and leaf chlorophyll content. Under CaCl_2_ stress, SiO_2_ NPs enhanced root growth, non-enzymatic antioxidant capacity, and antioxidant enzyme activities (catalase-CAT, ascorbate peroxidase-APX, and GR), while these improvements were not observed under NaCl and NaCl + CaCl_2_ stress. ZnO NPs caused greater physiological damage under CaCl_2_ and NaCl + CaCl_2_ stress compared to NaCl alone, suggesting that the interaction between ZnO NPs and CaCl_2_ impaired root development and water uptake, ultimately reducing PSII efficiency through oxidative damage. The synergistic effect between NPs and salinity stress was limited, observed only between SiO_2_ NP and CaCl_2_ stress in total flavonoid content. Overall, both NPs benefited hydroponic lettuce under non-saline conditions, with SiO_2_ NPs enhancing tolerance under CaCl_2_ stress, though their interaction with salinity as a eustressor was limited. These results suggest that SiO_2_ NPs enhance salinity tolerance in hydroponics, whereas ZnO NPs should be used with caution under saline conditions.

## 1 Introduction

Freshwater availability and quality for irrigation are essential for crop yield and sustainable agriculture. However, freshwater salinization, once considered a predominantly coastal issue, is now increasingly observed in inland regions due to a combination of human activities and natural processes (Jeppesen et al., 2020; Szklarek et al., 2022; Tuteja, 2007). These include rock weathering, high surface evaporation, prolonged drought periods, rising sea levels, poor water management in agriculture and industry, historical seawater intrusion, and runoff from deicing salts. For example, between 2006 and 2016, approximately 10 to 25 million tons of salt (predominantly NaCl) were applied annually for road deicing (Gopalakrishna and Gestwick, 2019), and arable land was salinized by seawater containing approximately 460 mM of Na^+^ and 540 mM of Cl^-^ with other ions (Mahajan and Tuteja, 2005). Salinity stress is a significant limiting factor for crop growth and productivity worldwide. Salinity stress restricts plant growth primarily through osmotic stress, which limits water uptake due to high salt concentrations near the roots, and ionic stress, which induces cellular injury and nutrient imbalances (Greenway and Munns, 1980). Both mechanisms trigger excessive production of reactive oxygen species (ROS) that disrupt cellular metabolism and lead to significant physiological, biochemical, and molecular impairments (Hasanuzzaman et al., 2021). To mitigate these adverse effects of salinity stress, researchers have introduced several strategies to improve plants’ salinity stress tolerance, such as conventional breeding, genetic engineering, soil amendments, and biostimulants, with ongoing efforts to develop new strategies (Choi et al., 2024; Farouk and Al-Huqail, 2022; Fita et al., 2015; Sofy et al., 2020).

Eustress is a beneficial form of stress that enhances plant responses such as growth, physiology, and antioxidant activity. Plants exposed to salinity stress often upregulate secondary metabolites, such as phenolic compounds and terpenoids, to enhance their defense systems, and these substances have also presented beneficial properties for human health, including anticancer, antioxidant, and anti-inflammatory activities (Kim et al., 2008). This has led to growing interest in eustressors for their potential to enhance health benefits by modulating physiological processes and defense mechanisms through controlled stress (Vázquez-Hernández et al., 2019). In addition, salinity stress has been shown to improve plant traits such as aroma and overall fruit quality, as well as antioxidant metabolites in various plant species (Rouphael et al., 2018). In many studies with salinity stress, NaCl is the predominant sole stress agent (Rouphael and Kyriacou, 2018). However, real-world salinity stress involves a complex mix of salts, including CaCl_2_ and others, highlighting the need for research not only on their stress effects but also on their potential roles as eustressors to better understand and represent natural environmental conditions.

Nanoparticles (NPs) are innovative tools that promote plant tolerance to abiotic stresses, effectively modulating key physiological, biochemical, and molecular processes. Nanotechnology explores materials sized between 1 and 100 nm in at least one dimension, where their nanoscale size and increased surface area provide unique properties that distinguish them from bulk materials, including enhanced electrical conductance, magnetism, chemical reactivity, optical effects, and physical strength (Jeevanandam et al., 2018). This technology has been investigated to address various agricultural challenges through applications such as nanoherbicides, nanofungicides, nanofertilizers, nanopesticides, and nanosensors, while using a variety of NPs, including iron (Fe), zinc (Zn), carbon, silver (Ag), chitosan, titanium (Ti), manganese (Mn), silicon (Si), selenium (Se), copper (Cu), etc., to mitigate salinity stress in plants (Etesami et al., 2021; Paramo et al., 2020). These NPs have been reported to significantly boost plant growth and tolerance to salinity stress by modulating key processes such as nutrient homeostasis, osmolyte biosynthesis, antioxidant defenses, photosynthesis, and phytohormone production (Khalid et al., 2022). However, excessive NP concentrations can cause phytotoxic effects, whereas insufficient levels often result in nonsignificant responses, with these outcomes varying by plant tissue, developmental stage, and species, highlighting the importance of optimizing NP application strategies (Lee and Leskovar, 2025; Zulfiqar and Ashraf, 2021).

Lettuce (*Lactuca sativa)*, although considered a salinity-sensitive species (Shannon and Grieve, 1998), has also been studied for the beneficial effects of salinity stress, such as enhanced antioxidant compounds in lettuce, but with reduced growth and yield (Garrido et al., 2014; Sakamoto et al., 2014). There is limited research on how different salts affect lettuce physiology, morphology, and biochemistry as both stressors and eustressors, and even less on how nanoparticles impact lettuce grown under various types of salt stress. To address these gaps, a hydroponic experiment was conducted to determine how NPs (SiO_2_ and ZnO) modulate lettuce growth and salinity tolerance, focusing on the physiological and biochemical changes under different salinity stress conditions.

## 2 Materials and methods

### 2.1 Plant materials and growth conditions

Green romaine lettuce ‘Green Forest’ (Johnny’s Selected Seeds, Winslow, ME, USA) was sown in 98-plug rockwool sheets (Grodan A-OK 3.81 cm × 3.81 cm Starter Plugs, Roermond, The Netherlands) and grown in a growth chamber (PGR15, Conviron Ltd., Winnipeg, Manitoba, Canada) set at 21 °C with a light intensity of 200 µmol m^-2^ s^-1^ (16/8 h, day/night). After germination, seedlings were fertigated daily with a nutrient solution containing 75 mg L^-1^ nitrogen (N) of Peter Professional 20% N-4.3% phosphorus (P)-16.6% potassium (K) (ICL Specialty Fertilizers, Tel Aviv, Israel) and transplanted into miniature deep-water culture (DWC) hydroponic systems 14 days after sowing.

The DWC systems were located in a customized growth chamber with environmental conditions controlled by a commercial air conditioner for temperature and a relay-controlled humidifier for relative humidity (RH). The temperature was maintained between 20-25°C and RH was kept above 50% (**Supplementary Figure 1**), which were monitored using a BME280 digital sensor module (Adafruit Industries, New York, NY, USA) connected to an Arduino UNO microcontroller (Arduino, Monza, Lombardy, Italy). The light was supplied by LED panels (RAZR4, Fluence, Austin, TX, USA) at an intensity of 230 µmol m^-2^ s^-1^ (16/8 h, day/night).

Two transplants were placed in plastic net cups (5.08 cm diameter), spaced 24 cm apart within each DWC tank (35.56 cm × 20.32 cm × 12.40 cm plastic container, Sterilite Corporation, Townsend, MA, CA, USA), with the tanks positioned next to each other. Each DWC tank was filled with 4 L of nutrient solution, continuously aerated at 3.9 L min^-1^ using an airstone connected to an air pump (Active Aqua AAPA 7.8 L, Hydrofarm, Shoemakersville, PA, USA). The nutrient solution was formulated using municipal water and Peters Professional 20% N-4.3% P-16.6% K fertilizer at a rate of 150 mg·L^-^¹ N, resulting in a final concentration of 10.71 mM N, 1.06 mM P, 3.18 mM K, 2.72 mM calcium (Ca), 0.19 mM sulfur (S), 17.35 µM boron (B), 13.43 µM Fe, 6.83 µM Mn, 5.74 µM zinc (Zn), 2.95 µM copper (Cu), and 0.78 µM molybdenum (Mo). The solution had an electrical conductivity (EC) of 1.5 mS·cm^-1^ and a pH of 5.8, which were monitored using a portable pH and EC meter (HI9812-51, Hanna Instruments, Woonsocket, RI, USA), and the entire nutrient solution was replaced weekly.

### 2.2 Experimental design and treatments

This study employed a randomized complete block design with four blocks and two plant replications per block (a total of eight plants per treatment). Treatments were factorial combinations of three nanoparticle water dispersions (US Research Nanomaterials, Inc., Houston, USA): distilled water (control), SiO_2_ (100 ppm; 30 nm), and ZnO (100 ppm; 30–40 nm); and four salinity stress conditions: distilled water (non-saline), 50 mM NaCl, 33.3 mM CaCl_2_, and a combination of 25 mM NaCl and 16.6 mM CaCl_2_ (NaCl + CaCl_2_). The NP concentrations were selected based on previous studies demonstrating that similar levels can promote plant growth and enhance tolerance to abiotic stress without inducing phytotoxic responses (Adrees et al., 2021; Asmat-Campos et al., 2022; Hasanaklou et al., 2023; Kumari et al., 2024). Similarly, the salt concentrations used for salinity stress were chosen based on studies indicating that comparable levels can elicit salinity eustress in lettuce without causing toxicity or irreversible damage (Garrido et al., 2014; Rouphael et al., 2018). Nanoparticle and salinity stress treatments were applied weekly on the same day as the nutrient solution renewal, starting three days after transplanting (DAT). Plant growth, physiological, and biochemical measurements were taken at 30 DAT.

### 2.3 Measurements

#### 2.3.1 Plant growth and morphology

Shoot fresh weight (FW), dry weight (DW), and root DW were measured; samples were oven-dried at 70°C for 72 h to measure DW. The shoot DW:FW ratio and root:shoot DW ratio were calculated.

Cumulative water usage was determined by calculating the weekly weight difference of the nutrient solution. Total root length, surface area, volume, and average root diameter were measured by scanning the washed whole root system using an Epson Perfection V700 PHOTO scanner (Epson America, Inc., Long Beach, CA, USA) and analyzing the root architecture with WinRHIZO Pro software (Regent Instrument Inc., Quebec City, Quebec, Canada).

The number of leaves was counted, and the total leaf area was measured using an LI-3100 Area Meter (LI-COR Biosciences, Lincoln, NE, USA). Leaf samples were flash-frozen with liquid nitrogen and lyophilized in a freeze-dryer (FreeZone, Labconco Corp., Kansas City, MO, USA). The samples were then ground into a fine powder using a paint shaker (Harbil, Wheeling, IL, USA) with 5 mm steel balls, and stored at -80 °C for later biochemical analyses.

#### 2.3.2 Leaf pigment and gas exchange rate

SPAD values were determined using a portable chlorophyll meter (SPAD-502 Plus, Konica Minolta, Tokyo, Japan) to estimate chlorophyll content in fully expanded mature leaves, with three readings taken per plant and the average value recorded. Additionally, chlorophyll *a*, *b*, and carotenoid concentrations were determined using the spectrophotometric method (Lichtenthaler and Wellburn, 1983). Lyophilized and ground leaf samples (10 mg) were homogenized with 1.5 mL of 80% acetone and centrifuged at 15,000 × *g* for 15 min. The absorbance of the supernatant was measured at 470, 646, and 663 nm using a microplate spectrophotometer (Multiskan GO, Thermo Scientific, Vantaa, Finland), and pigment concentrations were calculated as follows:


$$\text{Chlorophyll }a\;(\text{Chl }a)\;=\;12.21{\text{ A}}_{663}–\;2.81{\text{ A}}_{646}$$

$$\text{Chlorophyll }b\;(\text{Chl }b)\;=\;20.13{\text{ A}}_{646}–\;5.03{\text{ A}}_{663}$$

$$\text{Carotenoids }=\;(1000{\text{ A}}_{470}–\;3.27\text{ Chl }a–\;104\text{ Chl }b)\;/\;229$$

The net photosynthetic rate, transpiration rate, and stomatal conductance were measured in fully expanded mature leaves using a portable photosynthesis system (LI-6800; LI-COR Biosciences, Lincoln, NE, USA). Measurements were taken between 9 a.m. and 11 a.m., with the leaf chamber environment controlled by the sensor head under the following conditions: light intensity, 230 mmol m^−2^ s^−1^ (LED, 10% blue 90% red); CO_2_ concentration, 400 ppm; air flow rate, 500 mmol s^-1^. The electron transport rate was measured using a LI-600 porometer/fluorometer (LI-COR Biosciences, Lincoln, NE, USA).

#### 2.3.3 PSII efficiency, lipid peroxidation, and proline concentrations

Chlorophyll fluorescence was measured using fully mature leaves to assess the maximum quantum efficiency (Fv/Fm) and the effective quantum yield (Fq’/Fm’) of photosystem II (PSII) using a chlorophyll fluorometer (Op30p; Opti-Sciences Inc., Hudson, NH, USA) and the LI-600 porometer/fluorometer, respectively. Fv/Fm was measured after the leaves were dark-adapted for 30 min.

Lipid peroxidation in leaves was estimated by measuring malondialdehyde (MDA) concentration using a spectrophotometric method (Hodges et al., 1999; Loreto and Velikova, 2001). Lyophilized and ground leaf samples (25 mg) were homogenized with 1.5 mL of 0.1% (*w/v*) trichloroacetic acid (TCA) and extracted at 90 °C for 15 min. After centrifuging at 15,000 × *g* for 10 min, 200 µL of the supernatant was mixed with 800 µL of 20% TCA containing 0.5% (*w/v*) thiobarbituric acid (TBA). The mixture was incubated at 95 °C for 30 min, cooled on ice, and centrifuged at 15,000 × *g* for 10 min. A control mixture (-TBA), which excluded TBA from the final mixture (+TBA), was used to eliminate the interference from sugars and anthocyanins (Morales and Munne-Bosch, 2019). Absorbance was obtained at 440, 532, and 600 nm, and the MDA concentration was calculated as follows:


$$({\text{A}}_{532+\text{TBA}}-{\text{A}}_{600+\text{TBA}})-({\text{A}}_{532-\text{TBA}}-{\text{A}}_{600-\text{TBA}})=\text{a}$$

$$({\text{A}}_{440+\text{TBA}}-{\text{A}}_{600+\text{TBA}})\times 0.0571=\text{b}$$

$$\text{MDA equivalents}(\mu {\text{mol g}}^{-1})=([\text{a}-\text{b}]/157)\times 1000$$

Leaf proline concentrations was determined using a modified method of Bates et al. (1973). Lyophilized and ground leaf samples (25 mg) were homogenized with 1.5 mL of 3% (*w/v*) sulfosalicylic acid and centrifuged at 15,000 × *g* for 20 min. The supernatant (0.5 mL) was added with 0.5 mL of acetic acid and 0.5 mL of ninhydrin reagent (2.5% ninhydrin in 10.44 M glacial acetic acid and 2.4 M phosphoric acid). The reaction mixture was incubated at 95 °C for 1 h and vortexed after adding 1 mL of toluene. Absorbance was measured at 520 nm using toluene as the blank, and a standard curve was generated using proline.

#### 2.3.4 Non-enzymatic antioxidant capacity

Total phenolic content (TPC), total flavonoid content (TFC), 2,2′-Azinobis-(3-Ethylbenzthiazolin-6-Sulfonic Acid) (ABTS), and ferric reducing antioxidant power (FRAP) assays were conducted as described by Choi et al. (2024). Briefly, lyophilized and ground leaf samples (20 mg), collected from fully mature leaves, were homogenized and extracted with 1.4 mL of 80% methanol at 95 °C for 10 min. Following extraction, the samples were centrifuged at 15,000 × *g* for 15 min, and the resulting supernatant was used for the subsequent analyses.

TPC and TFC were determined using a modified method of Chandra et al. (2014). For the TPC assay, 10 μL of the sample extract was mixed with 100 μL of 2 N Folin-Ciocalteu reagent, followed by the addition of 90 μL of 7.5% sodium carbonate. After incubating at room temperature for 1 h, absorbance was measured at 735 nm, and a standard curve was generated with gallic acid. For the TFC assay, 20 μL of the sample extract was combined with 6 μL of 5% sodium nitrite, 12 μL of 10% aluminum chloride, 40 μL of 1 M sodium hydroxide, and 82 μL of distilled water. Absorbance was recorded at 515 nm and a standard curve was established with quercetin.

The ABTS assay was performed using a modified method of Re et al. (1999). A reaction mixture containing 7 mM ABTS and 2.45 mM potassium persulfate was incubated at room temperature for 16 h and subsequently diluted with methanol aiming the absorbance of 0.7 at 734 nm. The sample extract (10 μL) was added to the reaction mixture (190 μL) and absorbance was measured at 734 nm. A standard curve was generated using Trolox.

The FRAP assay followed a modified method of Benzie and Strain (1996). The sample extract (10 μL) was mixed with 300 μL of a solution containing 300 mM acetate buffer, 20 mM ferric chloride hexahydrate, and 10 mM TPTZ (2,4,6-Tris(2-pyridyl)-1,3,5-triazine) in a 10:1:1 ratio (*v/v/v*) in 40 mM hydrochloric acid. The control solution contained distilled water instead of TPTZ. Absorbance was recorded at 593 nm and a standard curve was created using Trolox.

The DPPH assay was performed using a modified method of Brand-Williams et al. (1995). The sample extract (10 μL) was combined with 190 μL of 200 μM DPPH (1,1-diphenyl-2-picrylhydrazyl) dissolved in ethanol and incubated at room temperature for 30 min. The absorbance of the DPPH free radical was measured at 515 nm and a standard curve was generated using ascorbic acid.

#### 2.3.5 Antioxidant enzyme activities

The activities of superoxide dismutase (SOD; enzyme commission number [EC] 1.15.1.1), guaiacol peroxidase (POD; EC 1.11.1.7), catalase (CAT; EC 1.11.1.6), ascorbate peroxidase (APX; EC 1.11.1.11), and glutathione reductase (GR; EC 1.8.1.7) were determined following the methods described by Lee et al. (2023). Briefly, lyophilized and ground leaf samples (40 mg), collected from fully mature leaves, were homogenized and extracted with 1.5 mL of 0.2 M potassium phosphate buffer (pH 7.8) containing 0.1 mM EDTA. The extracts were centrifuged at 15,000 × *g* for 20 min, and the resulting supernatant was used for the subsequent analyses.

SOD activity (Unit mg^-1^ protein) was determined using a modified method of Giannopolitis and Ries (1977). The enzyme extract (20 μL) was combined with 275 μL of a reaction mixture containing 50 mM phosphate buffer (pH 7.8), 2 mM EDTA, 9.9 mM L-methionine, 55 mM NBT, and 0.025% Triton X-100. The reaction was initiated by adding 5 μL of 146 mM riboflavin under LED light (7000 lux) for 5 min with tubes slowly oscillating at 1,400 rpm. Absorbance was recorded at 560 nm, and a standard curve was generated using SOD enzyme.

POD activity (Unit mg^-1^ protein) was measured using a modified method of Castillo et al. (1984). The enzyme extract (25 μL) was mixed with 250 μL of a reaction mixture containing 10 mM potassium phosphate buffer (pH 6.1), 96 mM guaiacol. The reaction was initiated by adding 25 μL of 12 mM hydrogen peroxide (H_2_O_2_). Absorbance was monitored at 470 nm for 3 min, and enzyme activity was calculated using the extinction coefficient of guaiacol (26.6 mM^-1^ cm^-1^).

CAT activity (Unit mg^-1^ protein) was assessed using a modified method of Hadwan (2018). The enzyme extract (30 μL) was mixed with 30 μL of 10 mM H_2_O_2_, followed by the addition of 300 μL of a solution containing 3.48 mM cobalt (II) nitrate hexahydrate, 0.82 mM sodium hexametaphosphate, and 96.4 mM sodium bicarbonate. Absorbance was recorded at 440 nm for 10 min, and enzyme activity was calculated using the extinction coefficient of H_2_O_2_ (43.6 mM^-1^ cm^-1^).

APX activity (Unit mg^-1^ protein) was determined using a modified method of Nakano and Asada (1981). The enzyme extract (30 μL) was added to 250 μL of a reaction mixture containing 50 mM potassium phosphate buffer (pH 7.0) and 0.5 mM ascorbate, and the reaction was initiated by adding 20 μL of 0.1 mM H_2_O_2_. Absorbance was measured at 290 nm for 3 min, and enzyme activity was calculated using the extinction coefficient of ascorbate (2.8 mM^-1^ cm^-1^).

GR activity (Unit mg^-1^ protein) was measured using a modified method of Smith et al. (1988). The enzyme extract (20 μL) was combined with 240 μL of reaction mixture containing 0.75 mM DTNB (5,5-dithiobis [2- nitrobenzoic acid]) and 0.1 mM NADPH. The reaction was initiated by adding 40 μL of oxidized glutathione (GSSG). Absorbance was measured at 412 nm for 3 min, and enzyme activity was calculated using the extinction coefficient of TNB (5- thio-2-nitrobenzoic acid) (14.15 mM^-1^ cm^-1^).

The total soluble protein content was measured using a modified method of Bradford (1976). The enzyme extract (10 μL) was added to 250 μL Bradford reagent, and absorbance was measured at 595 nm after incubating for 10 min. The standard curve was generated using bovine serum albumin.

### 2.4 Statistical analysis

All experimental data were subjected to a two-way analysis of variance (ANOVA) to examine the effects of salinity stress, nanoparticles, and their interaction using R software version 4.2.0 (R Development Core Team, 2022). Differences between treatment means were compared using Tukey’s HSD test at *P* ≤  0.05 with the ‘agricolae’ package (De Mendiburu, 2014). Pearson’s correlation coefficient method was used to evaluate the relationship between measurements with the ‘Hmisc’ package (Harrell and Dupont, 2020).

## 3 Results

### 3.1 Root and shoot growth

**Figure 1:**
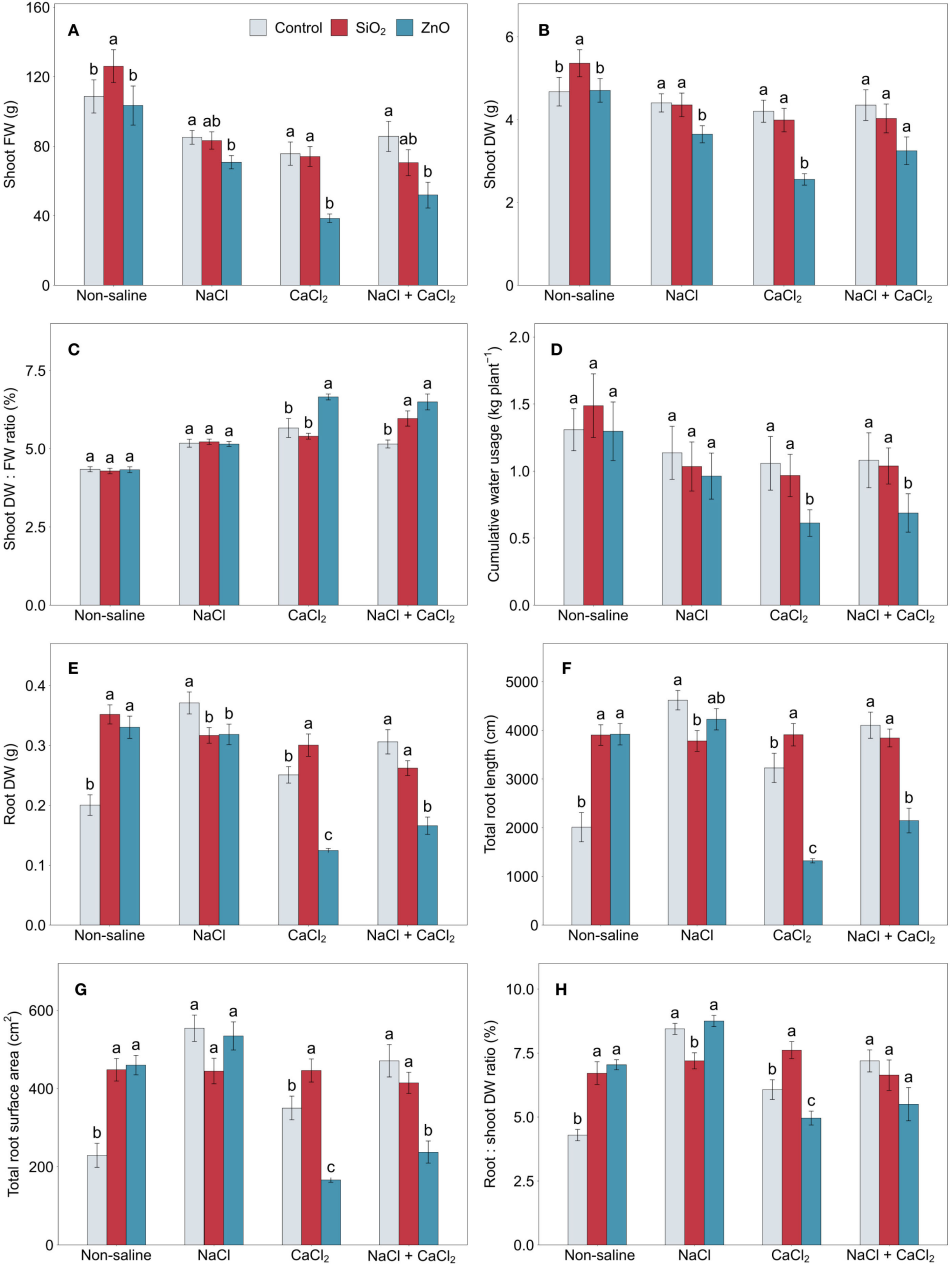
**(A)** shoot FW, **(B)** shoot DW, **(C)** shoot DW: FW ratio, **(D)** cumulative water usage, **(E)** root DW, **(F)** total root length, **(G)** total root surface area, and **(H)** root:shoot DW ratio in lettuce treated with nanoparticles under salinity stress. Different letters above the bars indicate statistically significant differences between nanoparticle treatments (control, SiO_2_, and ZnO) within each salinity stress (non-saline, NaCl, CaCl_2_, and NaCl + CaCl_2_) at *P* ≤ 0.05. Treatments sharing the same letter are not significantly different. Error bars indicate the standard error of the mean. FW, fresh weight; DW, dry weight.

In **Figures 1A, B**, under non-saline growth conditions, plants treated with SiO_2_ nanoparticles (NPs) showed greater shoot FW and DW compared to the control. However, no significant differences were observed between these groups under any salinity stress conditions. In contrast, ZnO NP-treated plants showed significantly or numerically reduced shoot FW and DW under all stress conditions. Additionally, ZnO NP-treated plants had higher shoot DW:FW ratios and lower cumulative water usage compared to the control under CaCl_2_ and NaCl + CaCl_2_ stress (**Figures 1C, D**).

In **Figures 1E–G**, SiO_2_ NP-treated plants showed increased root DW, total root length, and surface area compared to the control under non-saline and CaCl_2_ stress conditions while contrasting results were observed under NaCl stress with reduced root growth parameters. Similarly, ZnO NP-treated plants demonstrated enhanced root growth under non-saline conditions, while they were significantly reduced under CaCl_2_ and NaCl + CaCl_2_ stress conditions, along with reduced root DW under NaCl stress. Among all nanoparticle treatments, the root:shoot DW ratio in SiO_2_ NP-treated plants was the lowest under NaCl stress and the highest under CaCl_2_ stress (**Figure 1H**). The average root diameter was significantly higher in ZnO NP-treated plants compared to the control under CaCl_2_ stress (**Supplementary Figure 2A**), and total root volume (**Supplementary Figure 2B**) followed the treatment differences observed in total root surface area (**Figure 1G**).

In control plants without NPs, although shoot FW decreased under saline conditions compared to non-saline conditions, shoot DW showed no significant reduction under NaCl and NaCl + CaCl_2_ stress (**Figures 1A, B**). The root:shoot DW ratio of control plants was lowest under non-saline conditions compared to salinity stress conditions, which could be attributed to reduced root growth, including root DW, total root length, surface area, and volume (**Figures 1E–H**; **Supplementary Figure 2B**).

### 3.2 Leaf growth and physiology

**Figure 2:**
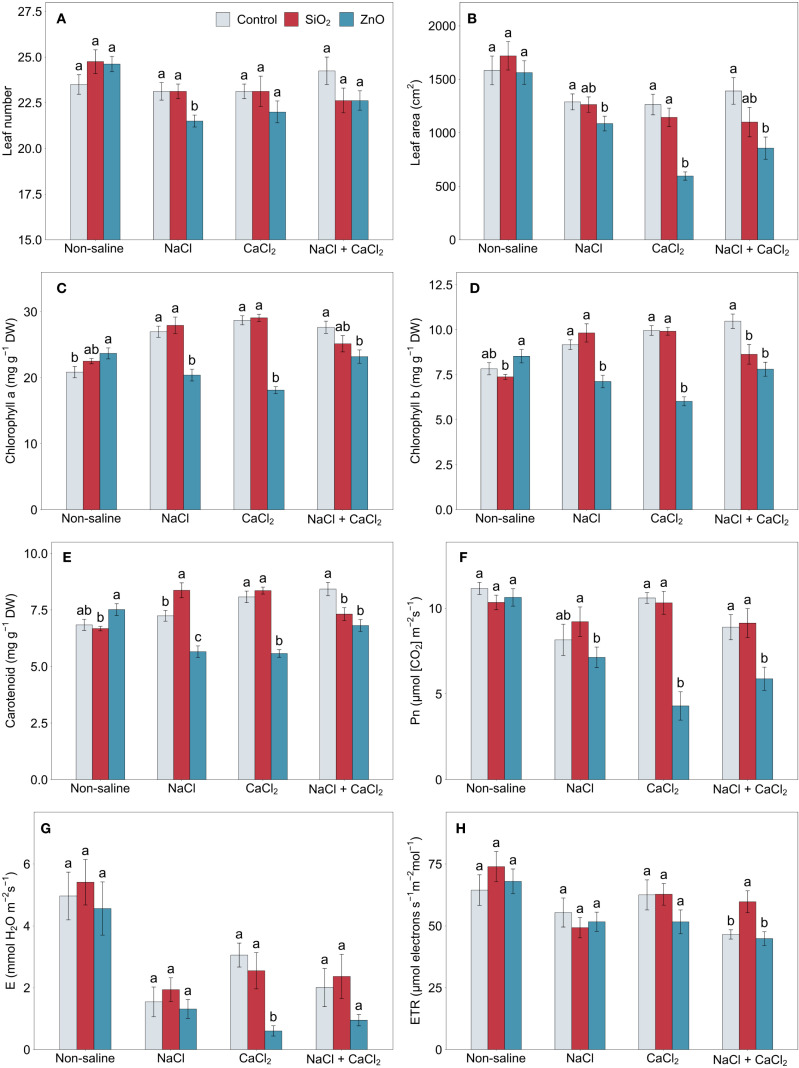
**(A)** leaf number, **(B)** leaf area, **(C)** chlorophyll a, **(D)** chlorophyll b, **(E)** carotenoid, **(F)** net photosynthetic rate, **(G)** transpiration rate, and **(H)** electron transport rate in lettuce treated with nanoparticles under salinity stress. Different letters above the bars indicate statistically significant differences between nanoparticle treatments (control, SiO_2_, and ZnO) within each salinity stress (non-saline, NaCl, CaCl_2_, and NaCl + CaCl_2_) at *P* ≤ 0.05. Treatments sharing the same letter are not significantly different. Error bars indicate the standard error of the mean; Pn, net photosynthetic rate. E, transpiration rate; ETR, electron transport rate.

ZnO NP-treated plants showed a reduced leaf number under NaCl stress and a smaller leaf area under all stress conditions compared to the control (**Figures 2A, B**). Chlorophyll *a* concentration was higher in ZnO NP-treated plants under non-saline conditions, but lower under all stress conditions, including chlorophyll *b* and carotenoid concentrations (**Figures 2C–E**). Carotenoid concentrations in control plants significantly increased under CaCl_2_ and NaCl + CaCl_2_ stress (*P* < 0.01). SiO_2_ NP-treated plants demonstrated higher carotenoid concentrations under NaCl stress compared to the control, but under NaCl + CaCl_2_ stress, chlorophyll *b* and carotenoid concentrations were lower. Differences in chlorophyll concentrations across treatments were generally aligned with SPAD values (**Supplementary Figure 3A**), except that SiO_2_ NP-treated plants had significantly higher SPAD than the control under CaCl_2_ stress. The net photosynthetic rate, transpiration rate, and stomatal conductance in ZnO NP-treated plants were significantly or numerically lower than those of the control under CaCl_2_ and NaCl + CaCl_2_ stress conditions (**Figures 2F, G**, **Supplementary Figure 3B**), while SiO_2_ NP-treated plants showed the highest electron transport rate among all treatments under NaCl + CaCl_2_ stress (**Figure 2H**).

### 3.3 Physiological and biochemical stress markers

**Figure 3:**
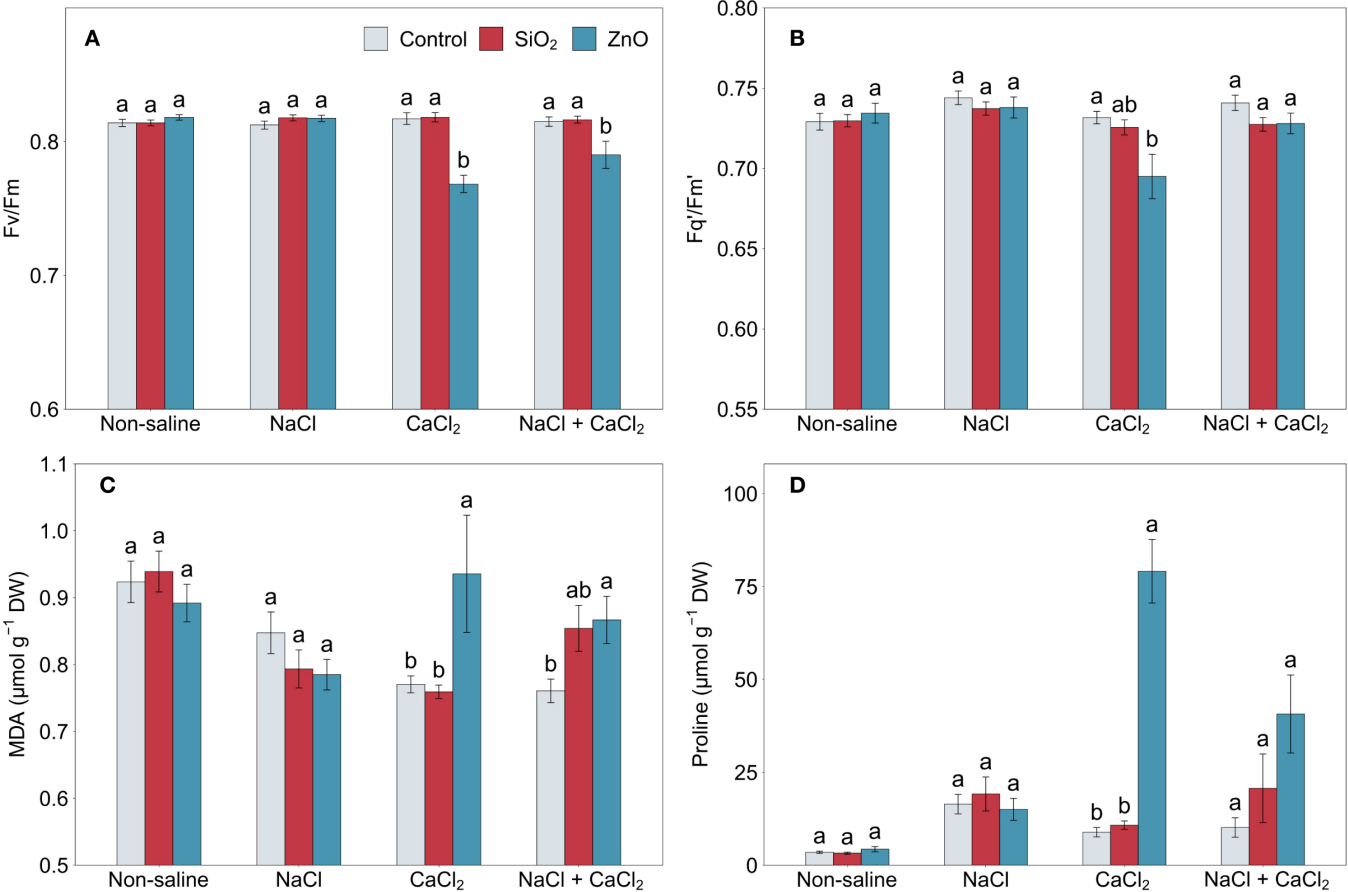
**(A)** Fv/Fm, **(B)** Fq’/Fm’, **(C)** MDA, and **(D)** proline in lettuce treated with nanoparticles under salinity stress. Different letters above the bars indicate statistically significant differences between nanoparticle treatments (control, SiO_2_, and ZnO) within each salinity stress (non-saline, NaCl, CaCl_2_, and NaCl + CaCl_2_) at *P* ≤ 0.05. Treatments sharing the same letter are not significantly different. Error bars indicate the standard error of the mean. MDA, malondialdehyde.

ZnO NP-treated plants showed reduced Fv/Fm under CaCl_2_ and NaCl + CaCl_2_ stress compared to the control (**Figure 3A**), as well as reduced Fq’/Fm’ under CaCl_2_ stress (**Figure 3B**). Leaf MDA concentration significantly increased in ZnO NP-treated plants under both CaCl_2_ and NaCl + CaCl_2_ stress conditions (**Figure 3C**). Proline concentration also increased significantly under CaCl_2_ stress and showed a numerical increase under NaCl + CaCl_2_ stress (**Figure 3D**). In contrast, no significant differences were observed in any of these parameters between SiO_2_ NP-treated plants and the control (**Figure 3**).

### 3.4 Non-enzymatic antioxidant capacity

**Figure 4:**
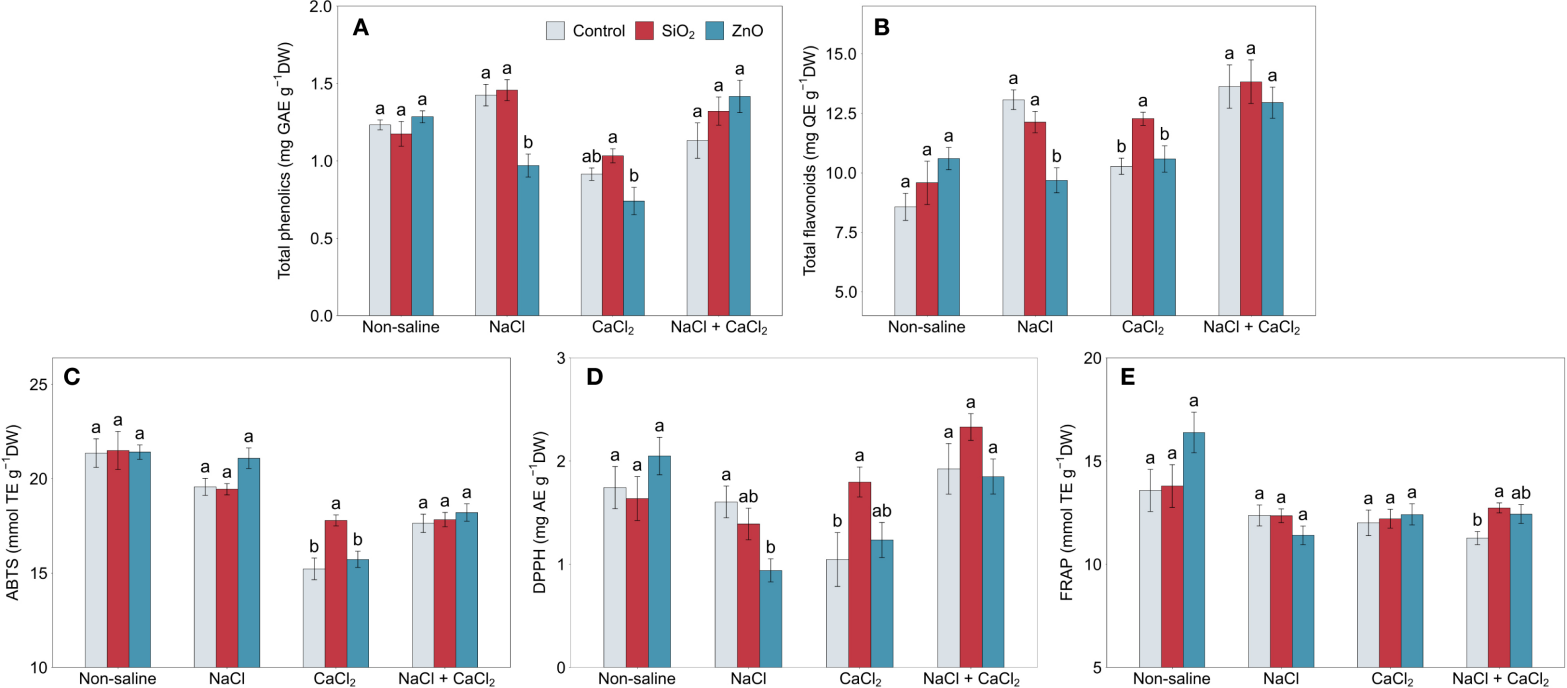
**(A)** total phenolics, **(B)** total flavonoids, **(C)** ABTS, **(D)** DPPH, and **(E)** FRAP in lettuce treated with nanoparticles under salinity stress. Different letters above the bars indicate statistically significant differences between nanoparticle treatments (control, SiO_2_, and ZnO) within each salinity stress (non-saline, NaCl, CaCl_2_, and NaCl + CaCl_2_) at *P* ≤ 0.05. Treatments sharing the same letter are not significantly different. Error bars indicate the standard error of the mean.

In **Figures 4A, B**, control plants showed a 15.6% increase in total phenolic content (TPC) under NaCl stress (*P* < 0.05) and increases of 52.5%, 19.9%, and 59.0% in total flavonoid content (TFC) under NaCl (*P* < 0.001), CaCl_2_ (*P* < 0.05), and NaCl + CaCl_2_ stress (*P* < 0.01), respectively, compared to non-saline conditions. Under NaCl stress, ZnO NP-treated plants demonstrated reduced total phenolic and flavonoid content and lower DPPH antioxidant capacity compared to the control (**Figures 4A, B, D**). In contrast, under CaCl_2_ stress, SiO_2_ NP-treated plants showed increased TFC, ABTS, and DPPH antioxidant capacities (**Figures 4B–D**) and higher FRAP under NaCl + CaCl_2_ stress compared to the control (**Figure 4E**).

### 3.5 Antioxidant enzyme activities

**Figure 5:**
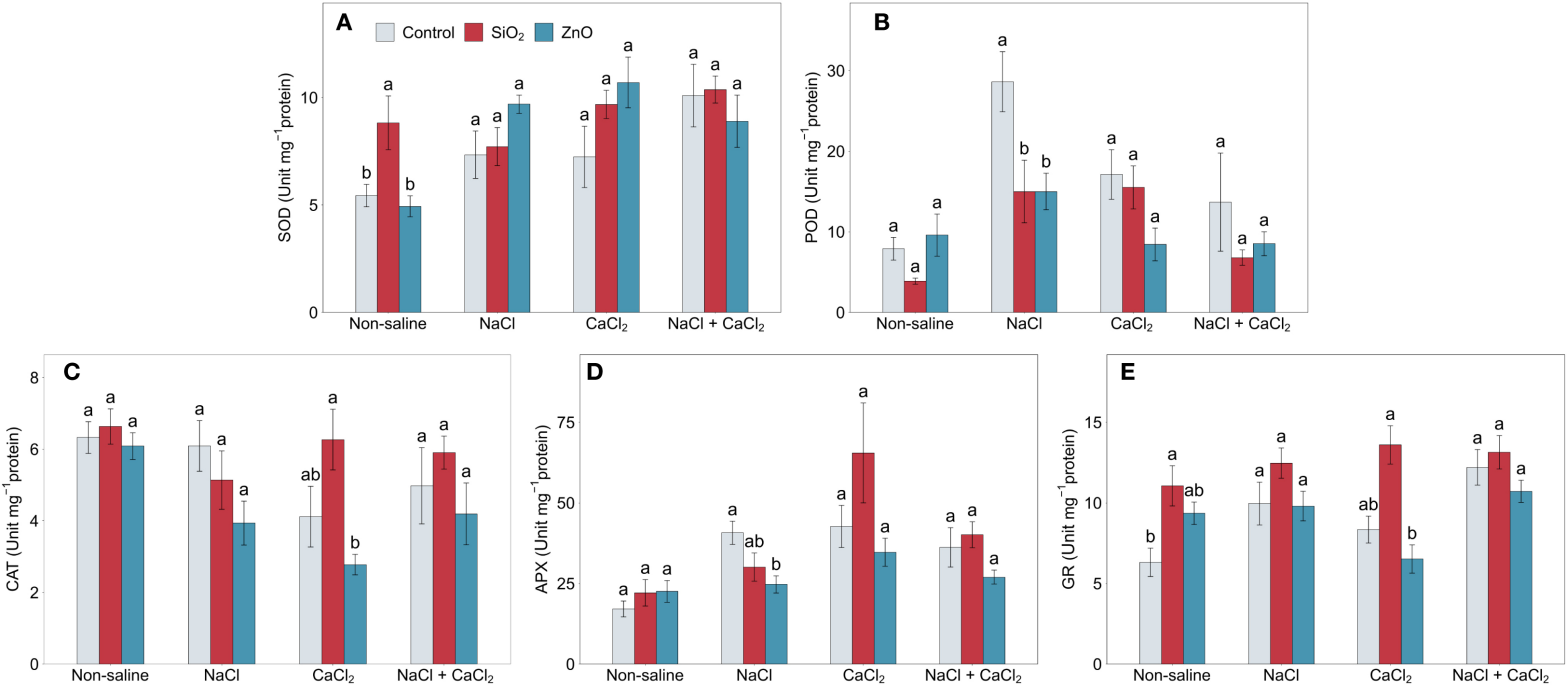
**(A)** SOD, **(B)** POD, **(C)** CAT, **(D)** APX, and **(E)** GR activities in lettuce treated with nanoparticles under salinity stress. Different letters above the bars indicate statistically significant differences between nanoparticle treatments (control, SiO_2_, and ZnO) within each salinity stress (non-saline, NaCl, CaCl_2_, and NaCl + CaCl_2_) at *P* ≤ 0.05. Treatments sharing the same letter are not significantly different. Error bars indicate the standard error of the mean. SOD, superoxide dismutase; POD, guaiacol peroxidase; CAT, catalase; APX, ascorbate peroxidase; GR, glutathione reductase.

Salinity stress significantly increased POD, APX, and GR activities in control plants (**Figure 5**). APX activity increased by over 100% and GR activity by over 50% under all salinity stress conditions compared to non-saline conditions, and POD activity increased by 262.9% and 116.9% under NaCl and CaCl_2_ stress (*P* < 0.05), respectively. Under non-saline conditions, SiO_2_ NP-treated plants showed higher SOD and GR activities compared to the control (**Figure 5A**). In SiO_2_ NP-treated plants, although POD activity was significantly lower under NaCl stress (**Figure 5B**), CAT, APX, and GR activities were numerically than in the control under CaCl_2_ stress (**Figures 5C–E**). In ZnO NP-treated plants, POD and APX activities were lower than those in the control under NaCl stress (**Figures 5B, D**), while no significant differences were observed for the other treatments in antioxidant enzyme activities.

### 3.6 Principal component analysis

**Figure 6:**
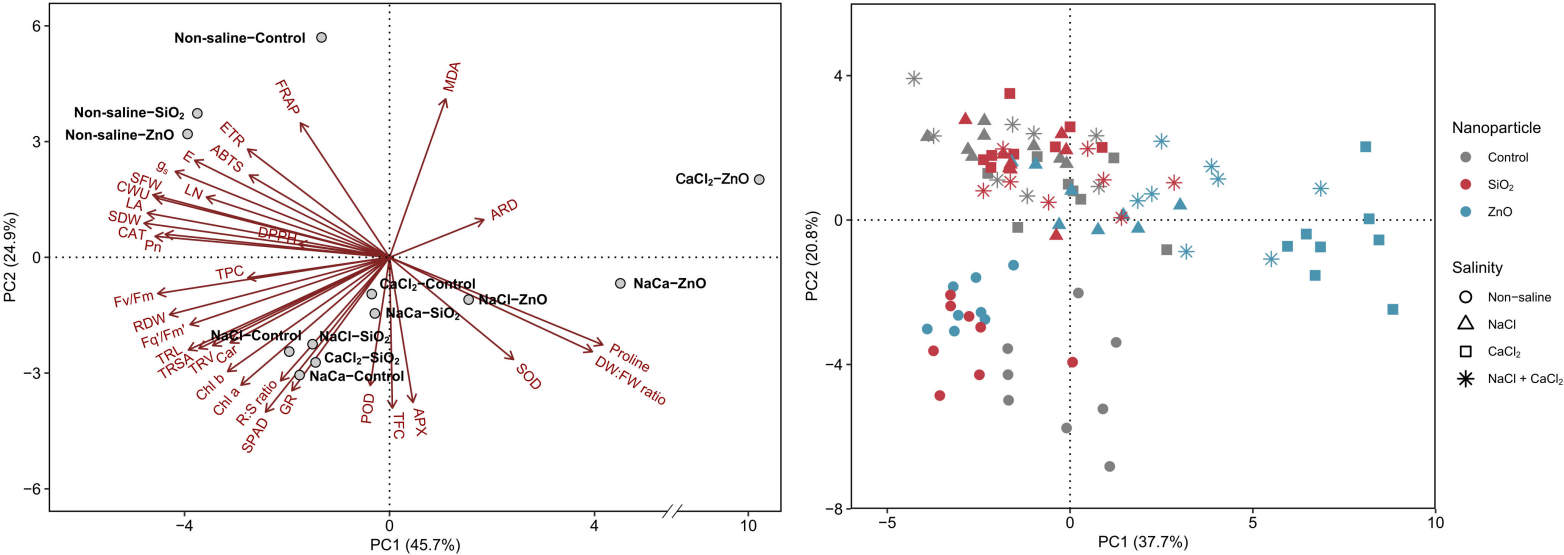
Principal component analysis (PCA) of growth and physiological parameters in lettuce treated with nanoparticles (control, SiO_2_, and ZnO) under salinity stress (non-saline, NaCl, CaCl_2_, and NaCl + CaCl_2_). NaCa, NaCl + CaCl_2_; MDA, malondialdehyde; ARD, average root density; DW:FW ratio, shoot DW:FW ratio; SOD, superoxide dismutase; APX, ascorbate peroxidase; TFC, total flavonoid content; POD, guaiacol peroxidase; GR, glutathione reductase; R:S ratio, root:shoot dry weight ratio; Chl, chlorophyll; Car, carotenoid; TRV, total root volume; TRSA, total root surface area; TRL, total root length; RDW, root dry weight; TPC, total phenolic content; Pn, net photosynthetic rate; CAT, catalase; SDW, shoot dry weight; LA, leaf area; CWU, cumulative water usage; SFW, shoot fresh weight; LN, leaf number; g_s_, stomatal conductance; E, transpiration rate; ETR, electron transport rate.

Plants grown under non-saline conditions clustered in the upper-left quadrant of the PCA biplot, characterized by high shoot biomass, leaf area, gas exchange rate, cumulative water usage, and electron transport rates (**Figure 6**). In contrast, SiO_2_ NP-treated and control plants under all salinity stress conditions clustered together in the lower-left quadrant, demonstrating higher TFC, leaf pigments, and antioxidant enzyme activities compared to those grown under non-saline conditions. ZnO NP-treated plants were distinctly separated from other treatment groups, particularly under CaCl_2_ and NaCl + CaCl_2_ stress. Key parameters associated with ZnO NP treatments included MDA, proline, average root diameter, and shoot DW:FW ratio, while most plant growth parameters were located opposite to their score points.

## 4 Discussion

### 4.1 Beneficial effects of nanoparticles under non-saline growth conditions

The application of nanoparticles in the hydroponic nutrient solution provided beneficial effects on lettuce growth under non-saline conditions (**Figure 6**). Plants treated with SiO_2_ NPs demonstrated increased shoot and root biomass, root system architecture, and antioxidant enzyme activities (SOD and GR) compared to the control (**Figures 1**, **5**; **Supplementary Figure 2**). Similarly, ZnO-NP treatments resulted in enhanced root biomass and architecture, with significant or numerical increases in leaf chlorophyll and carotenoid concentrations (**Figures 1**, **2**; **Supplementary Figure 2**). These findings align with those of Kumari et al. (2024) who reported that SiO_2_ NP treatment improved shoot and root biomass, total root length, and SOD and GR enzyme activities in maize. Also, ZnO NP application increased root system architecture and leaf chlorophyll concentrations in pearl millet (Tarafdar et al., 2014). The enhanced growth and physiological responses observed in lettuce confirm that SiO_2_ and ZnO NP treatments remain effective in hydroponic lettuce cultivation.

Plants treated with SiO_2_ and ZnO NPs demonstrated an increased root:shoot DW ratio compared to the control (**Figure 1H**), suggesting that their application stimulated more root growth relative to shoot growth. These NPs also increased shoot biomass (SiO_2_ NP) and leaf pigment content (ZnO NP). The enhanced shoot traits could be attributed to improved root system architecture, which likely enhanced nutrient uptake and ultimately influenced shoot development. However, it remains unclear whether the observed shoot responses were indirectly caused by root system improvements under NP application or directly resulted from the penetration and translocation of NPs within the shoot (Siddiqui et al., 2015).

### 4.2 Salinity stress responses of SiO_2_ nanoparticle-treated lettuce

Plants treated with SiO_2_ NPs partially retained the benefits of non-saline conditions under CaCl_2_ stress. Although no significant differences in shoot biomass were observed between SiO_2_ NP-treated plants and the control, root biomass and architecture remained significantly enhanced under CaCl_2_ stress (**Figure 1**; **Supplementary Figure 2**). SiO_2_ NP-treated plants demonstrated greater antioxidant capacity compared to control plants under CaCl_2_ stress, which may have contributed to sustaining improved root growth by reducing oxidative stress and maintaining cellular function; non-enzymatic antioxidant capacity, including TFC, ABTS, and DPPH, was significantly increased (**Figure 4**), and antioxidant enzyme activities, including CAT, APX, and GR, showed numerical increases with SiO_2_ NP treatments (**Figure 5**). These results suggest that SiO_2_ NP application effectively regulated stress response pathways by inducing antioxidant activities under CaCl_2_ stress conditions. It is speculated that SiO_2_ NP-treated plants were more tolerant to calcium (Ca^2+^) or chloride (Cl^-^) ion toxicity induced by CaCl_2_ stress compared to control plants.

Under NaCl stress, SiO_2_ NP-treated plants showed reduced root DW and total root length compared to the control (**Figures 1E, F**). Although their absolute root growth was similar to that observed under CaCl_2_ stress, control plants demonstrated a significant increase in root growth under NaCl stress compared to both non-saline and CaCl_2_ conditions, resulting in relatively lower root growth in SiO_2_ NP-treated plants under NaCl stress. The increased root growth in control plants could be a stress response mechanism compensating for reduced water and nutrient uptake, though further investigation is needed to clarify the underlying mechanism driving the NaCl stress-specific root growth spike compared to CaCl_2_ stress. Despite these differences in root growth, no significant differences were observed between SiO_2_ NP-treated and control plants in most physiological parameters, except for higher carotenoid concentrations and lower POD activity in SiO_2_ NP-treated plants (**Figures 2E**, **5B**), suggesting that SiO_2_ NP-treated plants were not necessarily more susceptible to NaCl stress than control plants. Although previous studies consistently reported beneficial effects of SiO_2_ NPs under NaCl stress, our results showed no significant improvement compared to the control, highlighting a rarely observed neutral response.

SiO_2_ NP-treated plants were unable to maintain the benefits of non-saline conditions under combined NaCl + CaCl_2_ stress, demonstrating a combination of tolerant and susceptible responses. Their leaf electron transport rate was higher than the control, indicating the upregulation of photoprotective mechanisms, along with an increased FRAP antioxidant capacity (**Figures 2H**, **4E**). However, leaf pigment concentrations were lower, and shoot DW: FW ratios were higher compared to the control (**Figures 1C**, **2D, E**), indicating reduced water retention under combined salinity stress. Furthermore, although statistically non-significant, SiO_2_ NP-treated plants demonstrated reduced plant biomass and increased leaf MDA concentrations compared to the control (**Figures 1A, B, E**, **3C**). These results suggest that SiO_2_ NP treatments did not provide adequate protection against physiological damage induced by NaCl + CaCl_2_ stress, although stress-induced responses were greater compared to the control. Given that all salinity stress treatments applied the same level of osmotic stress (derived from equivalent molar concentrations, as described in section 2.2), it is speculated that SiO_2_ NP treatments confer greater tolerance to Ca^2+^ or Cl^-^ ion toxicity but not to sodium (Na^+^) ion toxicity compared to the control. This differential effect on ion toxicity mitigation is supported by NP adsorption studies, such as Eissa (2024), which demonstrated distinct adsorption rates of calcium silicate NPs for different ions, such as Na^+^ and BO_3_^3-^.

### 4.3 Susceptible stress responses in ZnO nanoparticle-treated lettuce

Plants treated with ZnO NPs showed increased susceptibility to all salinity stress conditions compared to the control, which contrasts with findings under non-saline conditions (**Figure 6**). The growth and physiological damage were more pronounced under CaCl_2_ or NaCl + CaCl_2_ stress than under NaCl stress, particularly in plant biomass, leaf proline concentration, Fv/Fm, and Fq’/Fm’ (**Figures 1**, **3**). Fv/Fm and Fq’/Fm’ indicate the maximum and effective quantum efficiency of PSII, respectively, and are widely used as indicators of photosynthetic performance and plant stress tolerance (Baker and Oxborough, 2004). These findings sharply contrast with previous studies on sweet basil (Hasan et al., 2024), tobacco (Tirani et al., 2019), rice (Singh et al., 2022), and wheat (Adil et al., 2022), where root application of ZnO NPs enhanced physiological tolerance to salinity stress. Mustafa et al. (2024) reported that the application of ZnO NPs reduced pea grain yield under salinity stress, but the results were inconsistent across different NP and salinity concentrations. The discrepancies observed in this study suggest that the effects of NPs can vary significantly depending on plant species, nanoparticle characteristics, and stress intensity, while the contrasting results highlight the need to investigate which factors significantly influenced the transition from tolerance to phytotoxicity under salinity stress. In this regard, the present study lacks an evaluation of multiple NP concentrations; therefore, further research is needed to identify the phytotoxicity threshold under salinity stress conditions.

The results suggest that the interaction between ZnO NPs and CaCl_2_ stress adversely affected root development, impairing water uptake, while the resulting oxidative damage in PSII reduced photosynthetic efficiency. The observed photo-oxidative damage is attributed to elevated reactive oxygen species (ROS) levels, as indicated by increased MDA and proline concentrations. Although proline functions as a compatible osmolyte that scavenges ROS, its significant accumulation in this study indicates excessive ROS generation, which negatively correlated with most physiological parameters (**Supplementary Figure 4**). The adverse responses of ZnO NP-treated plants to CaCl_2_ and NaCl + CaCl_2_ stress clearly indicate that ZnO NP application is unsuitable under CaCl_2_ stress conditions for hydroponic lettuce cultivation.

Under NaCl stress, ZnO NP-treated plants showed no significant deleterious effects on water uptake, oxidative damage, or photosynthetic capacity compared to the control, while their antioxidant capacity was reduced with significantly lower TPC, TFC, DPPH capacity, and POD and APX enzyme activities (**Figures 4**, **5**). It is speculated that Na^+^ ion toxicity, induced by NaCl stress, may have antagonized the uptake of potassium (K^+^) ions more extensively with ZnO NP application, leading to an imbalanced K^+^/Na^+^ ratio that could inhibit antioxidant activities (Wakeel, 2013). Although Na^+^ and K^+^ concentrations were not measured, the reduced antioxidant activity with ZnO NP treatment under NaCl stress suggests the need for future studies on tissue-specific ion accumulation and K^+^/Na^+^ ratios.

The consistent physiological responses in ZnO NP-treated plants across varying salinity stress conditions, such as reduced plant biomass and leaf pigment content, indicate that these traits were specifically disrupted by the antagonistic interaction between ZnO NPs and osmotic stress. In contrast, salt-specific responses, such as increased proline concentration under CaCl_2_ stress, imply that these traits resulted from ZnO NPs exacerbating nutrient imbalance symptoms caused by specific ion toxicities. Also, the greater magnitude of stress damage observed under CaCl_2_ stress compared to NaCl + CaCl_2_ stress, reflected in traits such as root system architecture, Fq’/Fm’, and proline concentration (**Figures 1**, **3**; **Supplementary Figure 2**), suggests that the higher concentration of Ca^2+^ or Cl^-^ ions in CaCl_2_ stress was more detrimental to ZnO NP-treated plants than the combined stress conditions, which included added Na^+^ ions but lower Ca^2+^ or Cl^-^ ion concentrations.

### 4.4 Mechanisms driving nanoparticle phytotoxicity under salinity stress

The beneficial effects of SiO_2_ and ZnO NPs observed under non-saline conditions were reduced or even became deleterious when plants were subjected to salinity stress. One possible mechanism underlying these results is the increased aggregation of NPs under salinity stress. The ionic strength of the nutrient solution increases with the addition of salts, such as NaCl or CaCl_2_, which reduces electrostatic repulsion between particles (French et al., 2009). This, in turn, lowers the zeta potential (surface charge) of NP surfaces and promotes aggregation. In this study, increased NP aggregation within the nutrient solution could have decreased the number of functional NPs, thereby reducing the benefits observed under non-saline conditions. However, the negative effects of NPs, particularly in ZnO NP treatments, suggest that more complex mechanisms may be involved beyond the simple reduction of functional NPs.

Although the translocation of NPs to the shoot remains a controversial topic (Su et al., 2019), if NP aggregation occurs within plant tissues or if aggregates from the solution enter the root, these aggregates could cause mechanical cellular damage, thereby increasing NP phototoxicity. A study by Geisler-Lee et al. (2013) supports this mechanism, showing that Ag NPs were found aggregated at plasmodesmata in Arabidopsis roots. This could explain why ZnO NP application under salinity stress had more negative effects compared to SiO_2_ NPs because ZnO NPs are more likely to aggregate than SiO_2_ NPs under similar environmental conditions due to their higher van der Walls forces, which increase aggregate coefficients (Zhang et al., 2008). The greater susceptibility of ZnO NP-treated lettuce plants to CaCl_2_ stress, compared to NaCl stress, further supports the hypothesis that NP aggregation contributes to plant damage, because divalent ions like Ca^2+^ reduce electrostatic repulsion between NPs more effectively than monovalent ions such as Na^+^ (French et al., 2009), thereby promoting aggregation.

Another potential mechanism for the increased damage observed in ZnO NP-treated plants under salinity stress could be the increased adsorption of aggregated ZnO NPs to the root surface (Lin and Xing, 2008). ZnO NPs, with their positive zeta potential at pH 6, are more likely to adhere to the negatively charged root surface compared to SiO_2_ NPs, which have a negative zeta potential (Zhang et al., 2008). Spielman-Sun et al. (2019) reported that positively charged CeO_2_ NPs predominantly adhered to roots, while negatively charged NPs were more efficiently translocated from roots to shoots. The adsorption of NPs to the root surface can disrupt water and nutrient uptake (Khan et al., 2019), particularly under salinity stress, when osmotic pressure is already compromised, and this effect could be exacerbated when aggregated NPs block root pores. However, the interactions among nanoparticles, salinity stress, and plants remain poorly understood, highlighting the need for further mechanistic investigations involving nanoparticle characterization and localization using TEM or SEM microscopy.

### 4.5 Salinity stress as a eustressor to enhance nutraceutical quality

Our findings confirm that salinity stress can play a dual role in plant quality, acting both as a stressor that challenges plant growth and as a eustressor that stimulates the production of antioxidant-related compounds and enzyme activities, ultimately enhancing plant nutraceutical quality. The reduction in shoot FW under saline conditions in the control plants, with no significant reduction in shoot DW under NaCl and NaCl + CaCl_2_, suggests that salinity stress primarily affected water content rather than biomass accumulation. This is further supported by the higher shoot DW:FW ratio observed in control plants under salinity stress conditions, with no significant difference in cumulative water usage compared to non-saline conditions (**Figures 1C, D**).

Carotenoids, TPC, and TFC play key roles in oxidative stress mitigation, which likely explains their increased accumulation under salinity stress (**Figures 2**, **4**). Our results showed that carotenoid levels significantly increased under CaCl_2_ and NaCl + CaCl_2_, TPC increased under NaCl, and TFC increased across all salinity treatments. Similarly, salinity stress significantly elevated antioxidant enzyme activities, with APX and GR activities increasing by over 100% and 50%, respectively, under all salinity stress conditions, while POD activity showed significant increases under NaCl and CaCl_2_ stress (**Figure 5**). The enhanced bioactive compounds and enzyme activities were consistent with reports that salinity induces oxidative stress and activates enzymatic defense mechanisms to detoxify reactive oxygen species (Gill and Tuteja, 2010). These findings support the role of salinity stress in enhancing secondary metabolite biosynthesis and antioxidant enzyme activities as adaptive defense mechanisms (Rouphael and Kyriacou, 2018; Rouphael et al., 2018) and suggest that specific salt compositions differentially regulate secondary metabolism and oxidative stress responses.

Interestingly, the synergistic effects between NPs and salinity stress were minimal, observed only between SiO_2_ NP and CaCl_2_ in TFC (**Figure 4B**). These findings indicate that such interactions may vary with NP concentration, and salinity type and concentration, emphasizing the need for further research to optimize NP and salinity applications for enhancing plant nutraceutical quality. From a practical perspective, soil-based application of eustressors may be impractical due to the risk of overstressing plants and potential soil degradation, leading to persistent salinity stress. In contrast, soilless culture systems, with proper nutrient solution management, could provide a controlled strategy to enhance secondary metabolite production without compromising growth and yield.

## 5 Conclusion

This study expands our understanding of how SiO_2_ and ZnO nanoparticles (NPs) influence morphological, physiological, and biochemical responses in plants under different salinity stress conditions, with the goal of evaluating their potential to enhance salinity tolerance and nutraceutical quality in hydroponically grown lettuce. Our findings demonstrate that both SiO_2_ and ZnO NPs can improve lettuce growth under non-saline conditions, with SiO_2_ NPs enhancing biomass, root architecture, and antioxidant activities, while ZnO NPs promoted root development and chlorophyll content. Under salinity stress, not all NPs were effective. SiO_2_ NPs notably enhanced tolerance to CaCl_2_ stress by increasing root growth and antioxidant capacity, while ZnO NPs interacted negatively with CaCl_2_ stress, resulting in physiological damage and reduced PSII efficiency, highlighting the complex nature of NP-salinity interactions. Although salinity acted as a eustressor for secondary metabolite accumulation, its synergy with NPs was minimal, suggesting that further research is needed to clarify these interactions. The selective benefits of SiO_2_ NPs, particularly under CaCl_2_ stress, underscore their potential for enhancing salinity stress tolerance, while the adverse effects of ZnO NPs indicate the need for cautious application. These findings emphasize the importance of targeted application strategies when integrating nanotechnology into salinity management practices to improve crop growth and quality in controlled environment agriculture.

## Data Availability

The original contributions presented in the study are included in the article/**Supplementary Material**. Further inquiries can be directed to the corresponding author.
