# Supplementary material for: SiO2 and ZnO nanoparticles and salinity stress responses in hydroponic lettuce: selectivity, antagonism, and interactive dynamics
Source: Front Plant Sci. 2025 Sep 26;16:1634675. doi: 10.3389/fpls.2025.1634675 (PMC12511065; doi:10.3389/fpls.2025.1634675)
Supplement: Supplementary file 1 [file SupplementaryFile1.docx]

Supplementary Material

**Supplementary Table 1.** ANOVA table of measured parameters as influenced by salinity stress (S), nanoparticle (N), and their interactions. FW, fresh weight; DW, dry weight; CWU, cumulative water usage; TRL, total root length; TRSA, total root surface area; ARD, average root diameter; TRV, total root volume; R:S, root:shoot DW ratio; LN, leaf number; LA, leaf area; Chl, chlorophyll; Car, carotenoid; Pn, net photosynthetic rate; E, transpiration rate; g_s_, stomatal conductance; ETR, electron transport rate; MDA, malondialdehyde; TPC, total phenolic content; TFC, total flavonoid content; SOD, superoxide dismutase; POD, guaiacol peroxidase; CAT, catalase; APX, ascorbate peroxidase; GR, glutathione reductase.

|  | Shoot FW | Shoot DW | DW:FW | CWU | Root DW | TRL | TRSA | ARD | TRV | R:S | LN | LA |
| --- | --- | --- | --- | --- | --- | --- | --- | --- | --- | --- | --- | --- |
| S | *** | *** | *** | *** | *** | *** | *** | *** | *** | *** | *** | *** |
| N | *** | *** | *** | *** | *** | *** | *** | *** | * | † | * | *** |
| S × N | *** | *** | NS | *** | *** | *** | *** | NS | *** | * | *** | *** |
|  |  |  |  |  |  |  |  |  |  |  |  |  |
|  | Chl a | Chl b | Car | SPAD | Pn | E | g_s_ | ETR | Fv/Fm | Fq'/Fm' | MDA | Proline |
| S | *** | ** | * | *** | *** | *** | *** | *** | *** | *** | *** | *** |
| N | *** | *** | *** | *** | *** | *** | *** | NS | *** | * | NS | *** |
| S × N | NS | * | * | NS | *** | *** | *** | † | NS | NS | ** | NS |
|  |  |  |  |  |  |  |  |  |  |  |  |  |
|  | TPC | TFC | ABTS | DPPH | FRAP | SOD | POD | CAT | APX | GR |  |  |
| S | *** | *** | *** | *** | *** | *** | *** | ** | *** | ** |  |  |
| N | † | † | NS | NS | NS | † | ** | ** | * | *** |  |  |
| S × N | * | NS | NS | NS | NS | NS | NS | † | NS | NS |  |  |

†, *, ** and *** show significant differences at *P* ≤ 0.1, 0.05, 0.01, and 0.001, respectively; NS, not significant at *P* ≤ 0.1.

**
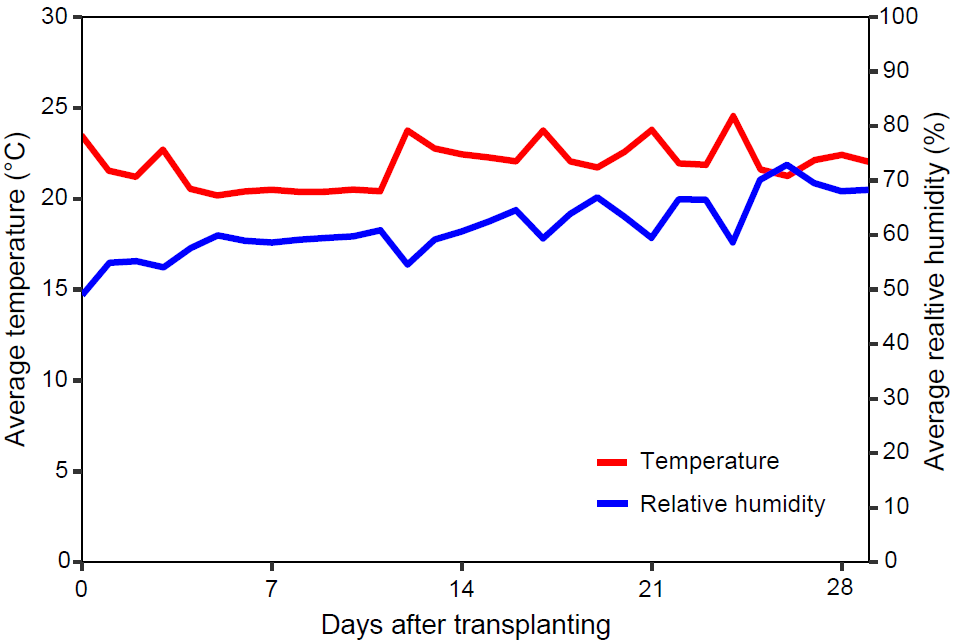
**

**Supplementary Figure 1.** Daily average temperature and relative humidity in growth chamber during the growth period.

**
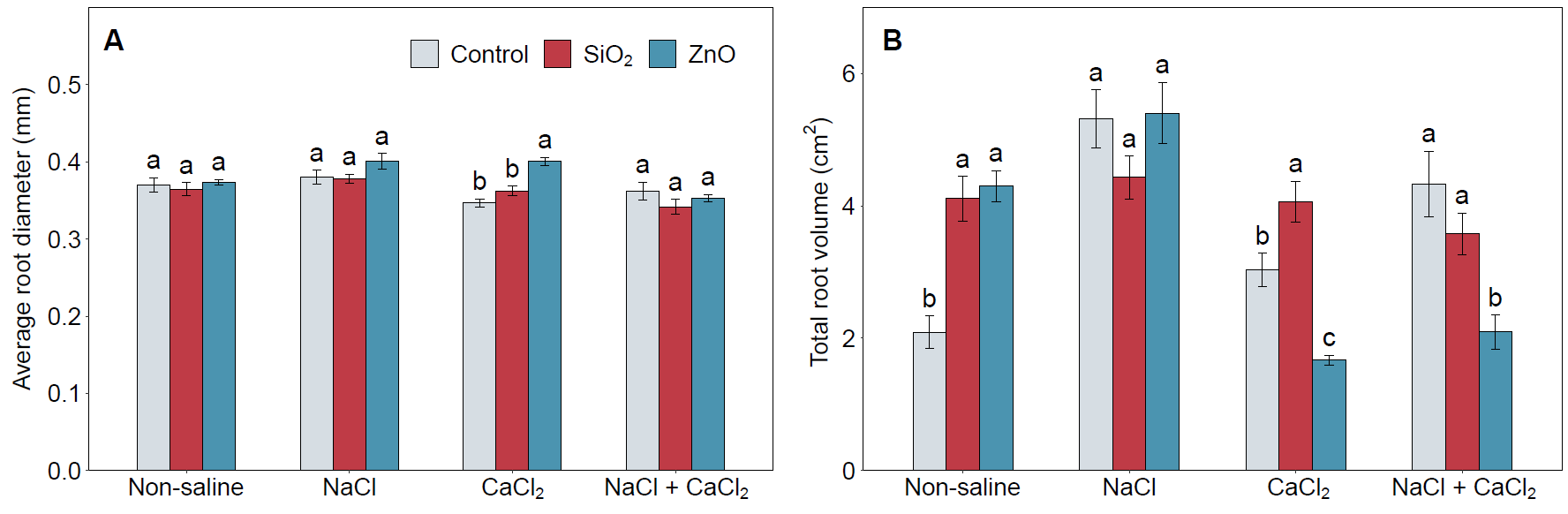
**

**Supplementary Figure 2.** **(A)** average root diameter and **(B)** total root volume in lettuce treated with nanoparticles under salinity stress. Different letters indicate significant differences between nanoparticle treatments (control, SiO_2_, and ZnO) within each salinity stress (non-saline, NaCl, CaCl_2_, and NaCl + CaCl_2_) at *P* ≤ 0.05. Error bars indicate the standard error of the mean.


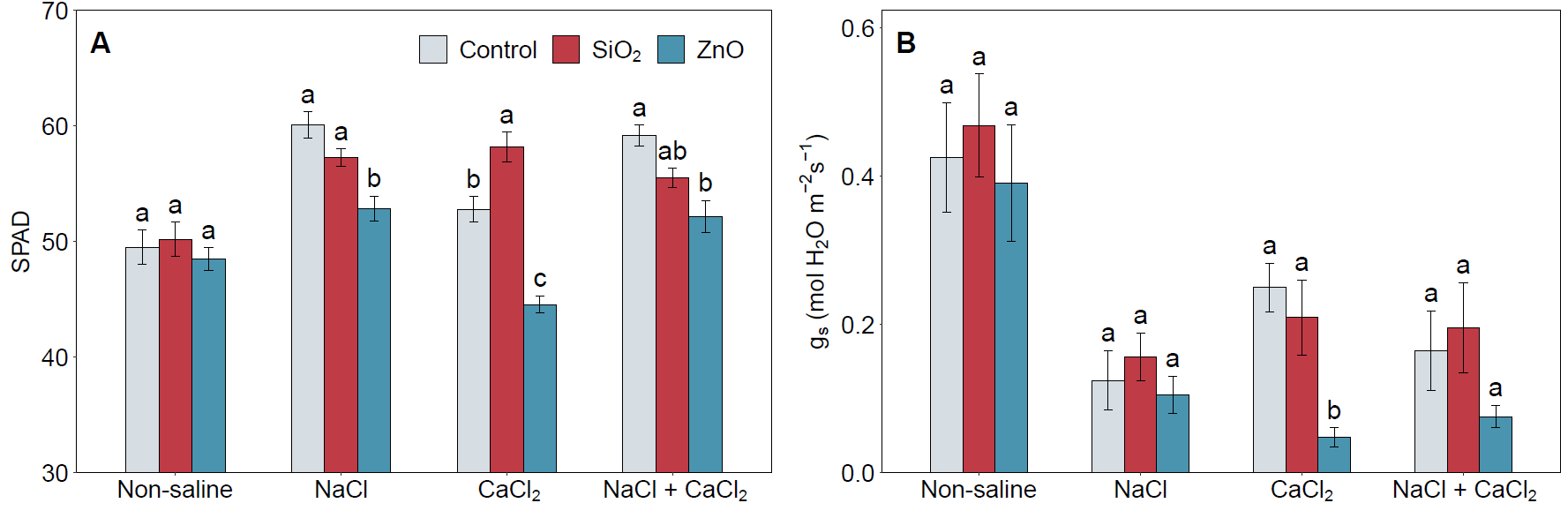


**Supplementary Figure 3.** **(A)** SPAD and **(B)** stomatal conductance in lettuce treated with nanoparticles under salinity stress. Different letters indicate significant differences between nanoparticle treatments (control, SiO_2_, and ZnO) within each salinity stress (non-saline, NaCl, CaCl_2_, and NaCl + CaCl_2_) at *P* ≤ 0.05. Error bars indicate the standard error of the mean. g_s_, stomatal conductance.


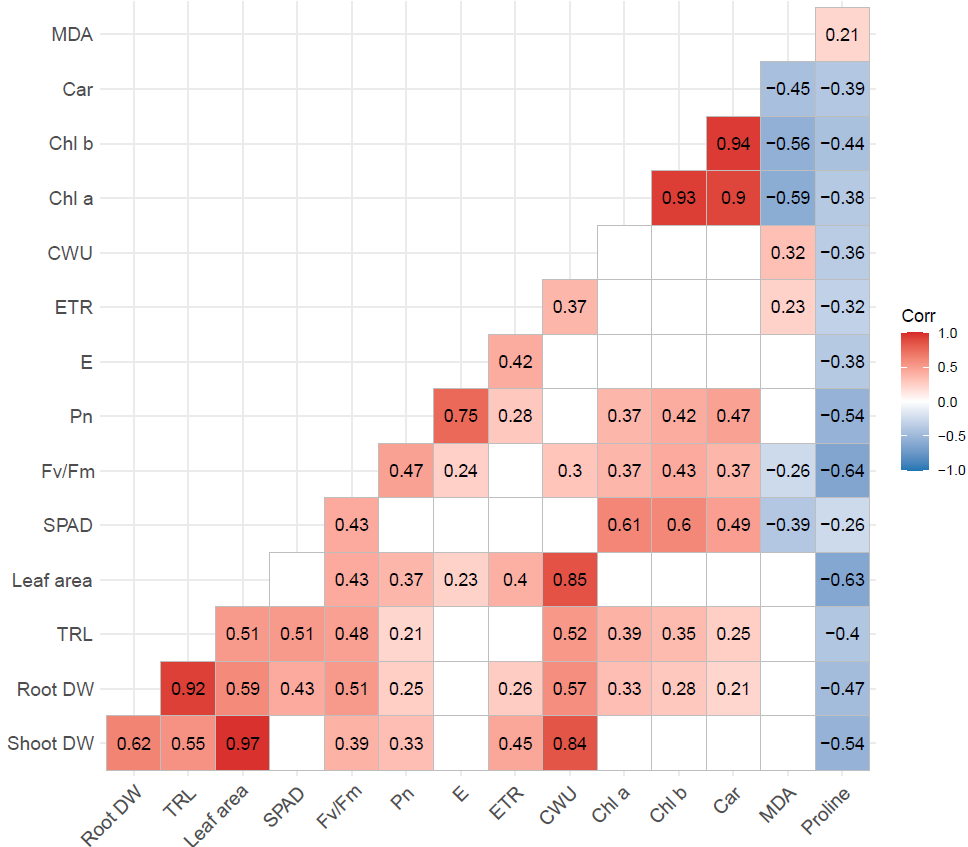


**Supplementary Figure 4.** Correlation between growth and physiological parameters in lettuce treated with nanoparticles (control, SiO_2_, and ZnO) under salinity stress (non-saline, NaCl, CaCl_2_, and NaCl + CaCl_2_). Only correlations with significance at *P* ≤ 0.05 are displayed. MDA, malondialdehyde; Car, carotenoid; Chl, chlorophyll; CWU, cumulative water usage; ETR, electron transport rate; E, transpiration rate; Pn, net photosynthetic rate; TRL, total root length; DW, dry weight.
